# Evaluating the Rehabilitation Needs of Stroke Patients in China: A Trend Analysis From 1990 to 2019

**DOI:** 10.1002/brb3.70389

**Published:** 2025-03-13

**Authors:** Peng Zhao, Huaxia Sun

**Affiliations:** ^1^ Department of Neurology The Second Affiliated Hospital of Hebei North University Zhangjiakou Hebei Province China; ^2^ Department of Neurology Weifang Hospital of Traditional Chinese Medicine Weifang Shandong Province China

**Keywords:** China, rehabilitation, stroke

## Abstract

**Background:**

Stroke remains a leading cause of death and long‐term disability in China. Rehabilitation is known to be an effective intervention for reducing disability among stroke survivors. This study seeks to quantify the rehabilitation needs of stroke patients in China by analyzing prevalence and years lived with disability (YLDs).

**Methods:**

To assess rehabilitation needs, we first estimated the prevalence and YLDs among stroke patients in China using data from the Global Burden of Disease Study 2019. The WHO Rehabilitation Need Estimator was then applied to these estimates to derive the rehabilitation requirements.

**Results:**

Our study reveals a substantial need for stroke rehabilitation services in China in 2019. An estimated 25.0 million stroke patients (95% uncertainty interval [UI] 22.0–28.0) could benefit from these services, accounting for 6.1 million years lived with disability (YLDs) [4.3–7.8]. This burden is disproportionately distributed between genders, with males experiencing 11.0 [9.5–12.0] million prevalent cases and 2.5 [1.8–3.2] million YLDs, compared to females with 14.0 [12.0–16.0] million cases and 3.6 [2.5–4.8] million YLDs. Worryingly, the age‐standardized prevalence of stroke in China has increased by 15.2% since 1990, rising from 11.2 per 1000 (95UI, 10.1–12.5) to 12.9 per 1000 (95UI, 11.5–14.4). Similarly, the age‐standardized YLD rate has grown by 15.8%, from 2.72 per 1000 (95UI, 1.94–3.49) to 3.15 per 1000 (95UI, 2.21–4.48). This upward trend in China contrasts sharply with the global picture, where age‐standardized prevalence and YLD rates have decreased by 6.2% and 4.8%, respectively.

**Conclusions:**

These findings demonstrate a critical need for expanded access to rehabilitation services within China's stroke care system. To enhance patient outcomes and address this growing need, increased investment in stroke rehabilitation infrastructure, training, and research is essential.

## Graphical abstract

In this study, we aim to quantify the rehabilitation needs of stroke patients in China by analyzing the prevalence and years lived with disability (YLDs). Age‐standardized stroke prevalence and disability burden (YLDs) in China have shown a slight increase from 1990 to 2019, affecting both men and women. Over the past three decades, the prevalence of stroke and its associated disability have demonstrated a modest upward trend. Our findings emphasize that this growing stroke burden in China underscores the ongoing need for strengthened prevention and rehabilitation strategies.

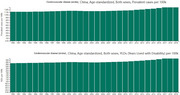

## 1 Introduction

Stroke is the leading cause of mortality and long‐term disability in China (Chao et al. 2021), placing immense pressure on the healthcare system and impacting millions of lives. While rehabilitation is a highly effective treatment for reducing disability in stroke patients (Dobkin 2005), access remains a significant challenge. It is estimated that only 40% to 70% of stroke survivors have access to these vital services, with the majority receiving inpatient treatment (Kam Yuet Wong et al. 2022). Furthermore, post‐discharge rehabilitation often lacks adequate professional guidance (Tu et al. 2023).

Stroke rehabilitation plays a crucial role in reducing disability and improving quality of life, yet a comprehensive assessment of the evolving rehabilitation needs in China is lacking. This study provides a detailed analysis of these needs, focusing on changes over the past three decades. Using data from the Global Burden of Disease Study 2019 (GBD 2019), we estimate prevalence, years lived with disability (YLDs), and assess the percentage change from 1990 to 2019

## 2 Methods

This study utilized data from the GBD Study 2019, as detailed in previous publications (Cieza et al. 2020; Feigin et al. 2021). The WHO Rehabilitation Need Estimator (available at https://vizhub.healthdata.org/rehabilitation/) was employed to calculate the prevalence and YLDs associated with stroke. Specifically, we selected “China and Global” in the “Location” section and “Cerebrovascular disease (stroke)” in the “Condition” section. This study was approved by the Ethics Committee of the Second Affiliated Hospital of Hebei North University, with a waiver of informed consent granted.

Our analysis included data for all ages and both sexes, encompassing the number of prevalent stroke cases and the prevalence per 1000 individuals. Age‐standardized prevalence and YLD rates were also calculated.

Following the GBD methodology, uncertainty was estimated by generating 1000 draws for each metric. The 95% uncertainty interval (UI) was then constructed using the 2.5th and 97.5th percentiles of these draws.

Percentage change from 1990 to 2019 was calculated as: (2019 value − 1990 value)/1990 value × 100%.

## 3 Results

In 2019, an estimated 25.0 million people in China (95%UI 22.0–28.0) needed stroke rehabilitation services, resulting in 6.1 million YLDs [4.3–7.8]. This burden was distributed across genders, with 11.0 million males [9.5–12.0] and 14.0 million females [12.0–16.0] requiring rehabilitation, contributing to 2.5 [1.8–3.2] and 3.6 [2.5–4.8] million YLDs, respectively (see Table 1).

**TABLE 1:** Global and China prevalence and years of life lived with disability for stroke patients in need of rehabilitation, all‐age counts, and age‐standardized rates for 1990 and 2019.

		Prevalence				Years of life lived with disability			
		All ages (millions)		Age‐standardized rate (per 1000)		All ages (millions)		Age‐standardized rate (per 1000)	
		2019	1990	2019	1990	2019	1990	2019	1990
China	ALL	25 (22–28)	10 (9–11)	12.9 (11.5–14.4)	11.2 (10.1–12.5)	6.1 (4.3–7.8)	2.4 (1.7–3.1)	3.15 (2.21–4.08)	2.72 (1.94–3.49)
	Male	11 (9.5–12)	4.3 (4.9–4.8)	11.2 (10.0–12.7)	9.53 (8.63–10.6)	2.5 (1.8–3.2)	0.99 (0.71–1.3)	2.64 (1.88–3.43)	2.22 (1.59–2.87)
	Female	14 (12–16)	5.7 (5.1–6.3)	14.3 (12.6–16.0)	12.6 (11.3–14.0)	3.6 (2.5–4.8)	1.4 (1.0–1.8)	3.58 (2.49–4.61)	3.13 (2.23–4.04)
Global	ALL	86 (79–94)	46 (42–50)	10.5 (9.66–11.5)	11.2 (10.3–12.2)	18 (13–23)	9.4 (6.8–12.0)	2.18 (1.57–2.77)	2.29 (1.65–2.91)
	Male	37 (34–41)	19 (18–21)	9.52 (8.71–10.4)	9.92 (9.05–10.9)	7.3 (5.3–9.3)	3.7 (2.7–4.7)	1.88 (1.36–2.40)	1.92 (1.39–2.45)
	Female	49 (45–53)	27 (25–29)	11.4 (10.5–12.4)	12.2 (11.3–13.3)	10.0 (7.5–13.0)	5.7 (4.1–7.2)	2.44 (1.75–3.08)	2.59 (1.87–3.28)

Alarmingly, the need for stroke rehabilitation has dramatically increased over the past three decades. From 1990 to 2019, the number of prevalent cases needing rehabilitation surged by 150.0%—a growth rate far exceeding the global average (150.0% vs. 87.0%). This increase was observed in both males (155.8%) and females (145.6%). Similarly, YLDs related to stroke increased by 154.2% in China, compared to 91.5% globally, with comparable increases seen in both genders (see Table 2).

**TABLE 2:** The percentage change of prevalent cases and years of life lived with disability of stroke contributing to the need for rehabilitation services from 1990 to 2019 in China and the Global.

		Prevalence (%)		Years of life lived with disability (%)	
		All age	Age‐standardized rate	All age	Age‐standardized rate
China	ALL	150.0	15.2	154.2	15.8
	Male	155.8	17.5	152.5	18.9
	Female	145.6	13.5	157.1	14.4
Global	ALL	87.0	−6.2	91.5	−4.8
	Male	94.7	−4.0	102.7	−2.1
	Female	81.5	−6.6	75.4	−5.8

**FIGURE 1:**
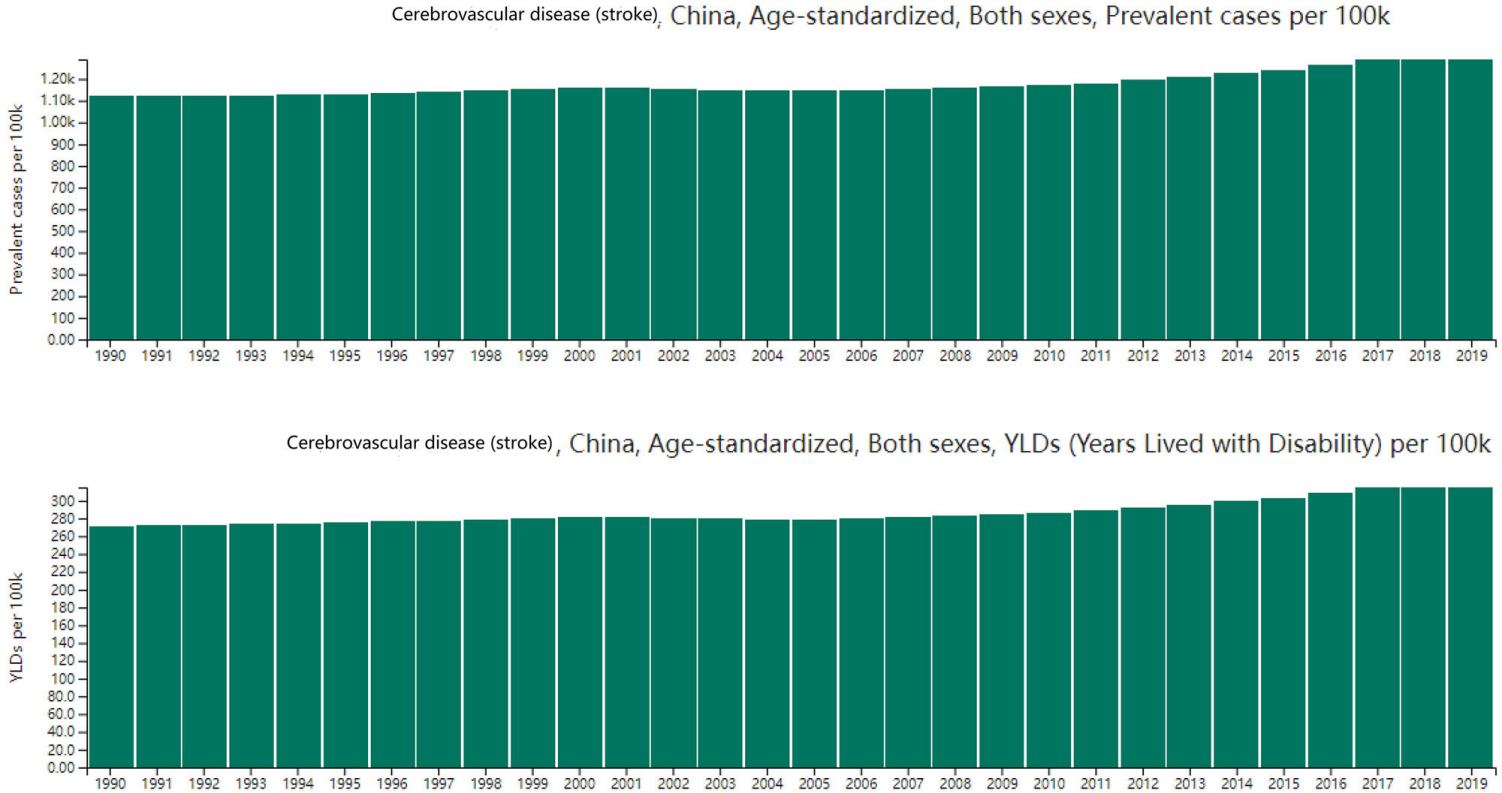
The age‐standardized prevalence and years of life lived with disability (YLDs) rates of stroke contributed to the need for rehabilitation services per 100k from 1990 to 2019.

Worryingly, age‐standardized prevalence and YLD rates in China have also climbed steadily since 1990. The prevalence rate rose from 11.2 per 1000 (95UI, 10.1–12.5) to 12.9 per 1000 (95UI, 11.5–14.4), marking a 15.2% increase. Similarly, the YLD rate increased by 15.8%, from 2.72 per 1000 (95UI, 1.94–3.49) to 3.15 per 1000 (95UI, 2.21–4.48). This upward trend was observed for both males (17.5% increase in prevalence, 18.9% increase in YLDs) and females (13.5% and 14.4%, respectively) (see Table 2 and Figure 1).

This rise in China stands in stark contrast to the global trend. Worldwide, age‐standardized prevalence and YLD rates have seen modest declines of 6.2% and 4.8%, respectively, since 1990, with both genders experiencing similar downward trends (see Table 2).

Furthermore, China's contribution to the global burden of stroke has grown significantly over the past three decades. Its share of global prevalence has risen from 21.7% to 29.1%, while its share of global YLDs has climbed from 25.5% to 33.9%. Within China, the proportion of stroke cases requiring rehabilitation has increased from 3.7% to 5.4%, and the proportion of YLDs attributable to stroke requiring rehabilitation has risen from 6.7% to 9.7%. These figures paint a clear picture: The demand for stroke rehabilitation services in China is not only immense but also continues to grow, indicating that the peak of this burden has yet to be reached.

**FIGURE 2:**
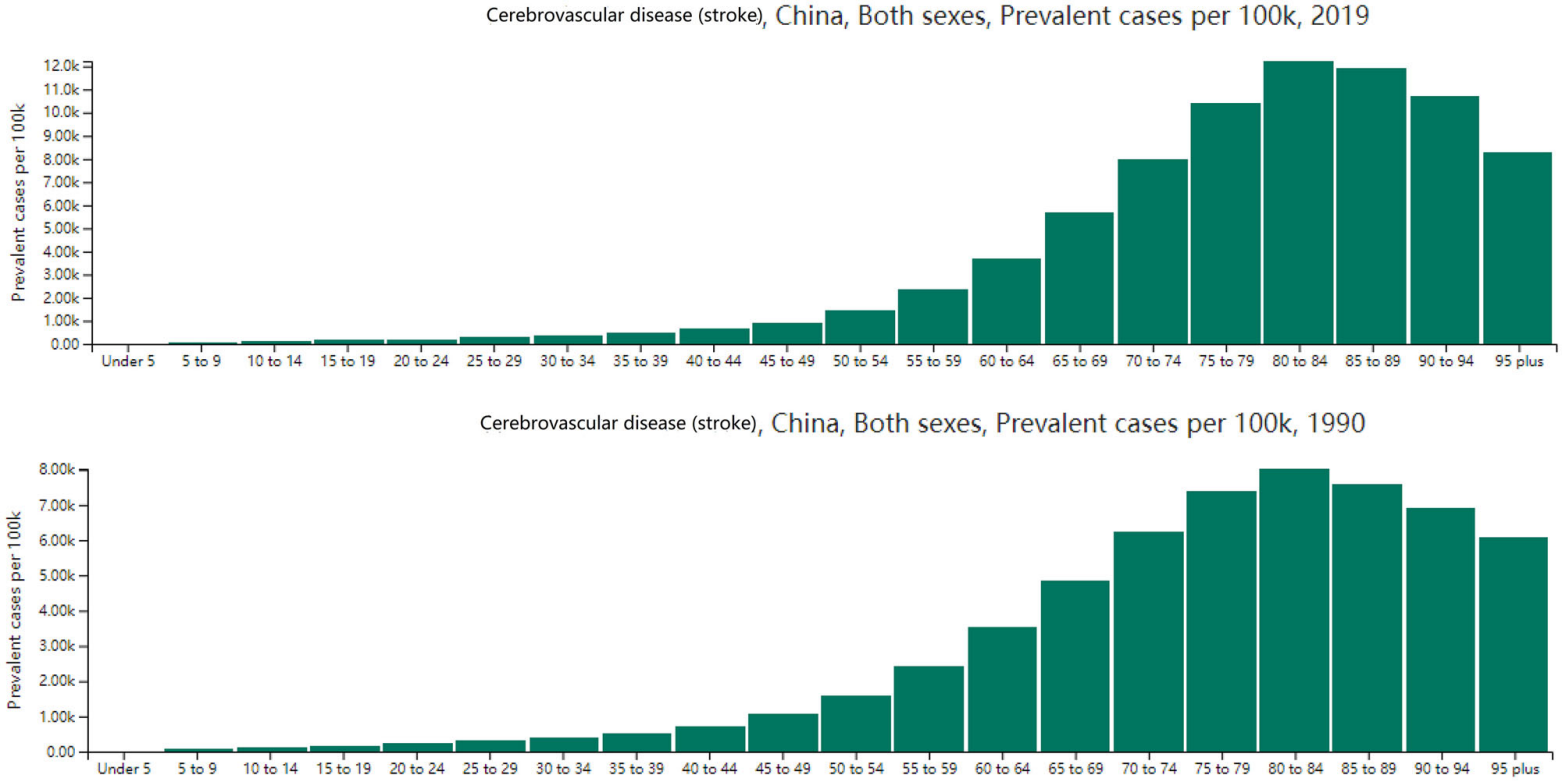
The prevalent stroke cases contribute to the need for rehabilitation services per 100k in different age groups.

Figure 2 illustrates the age‐specific prevalence of stroke per 100,000 population. Notably, the prevalence in 2019 shows a significant increase in the older age groups (65+) compared to 1990. This trend likely reflects improved survival rates among older stroke patients, leading to a larger population living with the long‐term effects of stroke and requiring rehabilitation.

## 4 Discussion

Globally, age‐standardized prevalence and YLD rates have shown modest declines since 1990, suggesting that the overall increase in stroke cases is largely attributable to population growth and aging (Cieza et al. 2020). However, China presents a different picture. Both the number of stroke cases and age‐standardized rates have risen, indicating a concerning increase in stroke incidence beyond what can be explained by demographic shifts. This trend is further evidenced by the consistent growth in stroke prevalence observed in China over the past decade (Tu et al. 2022). With China's aging population projected to peak in the next 20 years (Tu et al. 2022), the demand for stroke rehabilitation services is poised to escalate dramatically, placing an even greater strain on the healthcare system.

Despite this escalating need, access to timely stroke rehabilitation in China remains severely limited. Studies reveal a concerning gap between need and provision: Only 11.5% of stroke patients receive rehabilitation within 1 week of onset, a crucial window for recovery, and a staggering 42.4% receive no rehabilitation at all (Asakawa et al. 2017). This inadequacy is further underscored by research from Bettger et al. (2017), which found suboptimal rehabilitation assessment rates among acute ischemic stroke (AIS) patients in China, with significant variability across 219 hospitals (ranging from 41.4% to 81.5%).

This starkly contrasts with stroke rehabilitation rates in other developed countries. In the United States, for instance, participation in outpatient stroke rehabilitation increased from 27.2% in 2013 to 35.5% in 2015 (Ayala et al. 2018). Japan demonstrates even greater success with its long‐term care system for stroke, where 73.0% of patients receive rehabilitation treatment (Kinoshita et al. 2022). Similarly, in Germany, approximately half of all stroke patients receive specialized neurological or geriatric rehabilitation care (De Peretti et al. 2018). These comparisons highlight the potential for improvement in China's stroke rehabilitation services and underscore the need for increased investment and systemic change.

It is crucial to recognize that stroke impacts men and women differently. Women generally experience worse outcomes after stroke compared to men (Phan et al. 2017), highlighting the need for gender‐specific considerations in stroke management and rehabilitation (Glader et al. 2003). An extensive international trial (*n* = 19,652) found that while women with ischemic stroke had better survival rates, they were also more likely to experience greater disability and poorer quality of life after stroke (Carcel et al. 2019). This underscores the importance of tailoring rehabilitation programs to address the unique needs and challenges faced by women. Further research is needed to explore sex‐specific factors, such as functional cerebral asymmetries, to optimize stroke management and improve outcomes for both men and women (Bonkhoff et al. 2021).

Our study revealed a noteworthy trend: The percentage change in age‐standardized prevalence and YLDs was more pronounced among Chinese male stroke patients compared to females. This contrasts with the global pattern, where reduction rates were lower for males than for females. This finding highlights the need to pay particular attention to the needs of male stroke patients in China, who appear to be experiencing a disproportionate increase in stroke burden. As gender equity gains increasing prominence in healthcare discussions worldwide (McInnes et al. 2008; Volpe et al. 2023), it is essential to ensure that stroke rehabilitation services are accessible and effective for both men and women, addressing the unique needs and challenges faced by each gender (Tu and Wei 2024).

This study focuses solely on prevalence and YLDs to estimate rehabilitation needs in China. It does not address the specific types of rehabilitation required, the availability of such services, or their quality. While demand for rehabilitation is high, early rehabilitation services in China are inadequate (Asakawa et al. 2017). For example, studies have shown that only 11.5% of stroke patients receive rehabilitation within 1 week, and 42.4% receive no rehabilitation at all (Asakawa et al. 2017; Liu et al. 2020). In addition, in this study we found that the prevalence in 2019 shows a significant increase in the older age groups (65+) compared to 1990. Several factors likely contributed to this outcome. First, acute stroke treatments, such as intravenous thrombolysis (IVT) and endovascular therapy (EVT), are becoming increasingly prevalent. A recent study demonstrated a significant increase in IVT and EVT rates for AIS in China between 2019 and 2020, with overall rates of 5.64% for IVT and 1.45% for EVT (Ye et al. 2022). Additionally, effective control of stroke risk factors such as hypertension and diabetes has reduced the likelihood of stroke recurrence (Wu et al. 2019). Finally, overall life expectancy has increased (Chen et al. 2020).

## 5 Conclusions

This study provides the first comprehensive estimate of stroke rehabilitation needs in China, highlighting the substantial burden faced by the country. Our findings reveal that nearly one‐third of stroke patients requiring rehabilitation worldwide are in China, with a dramatic increase in cases over the past three decades. This underscores the urgent need for increased investment in rehabilitation infrastructure, training, and research, and for the integration of these services into the national stroke care system.

## Author Contributions

**Peng Zhao**: conceptualization, investigation, funding acquisition, writing – original draft, visualization, validation, methodology, software, formal analysis. **Huaxia Sun**: conceptualization, investigation, funding acquisition, writing – review and editing, visualization, validation, project administration, resources, supervision, data curation.

## Conflicts of Interest

The authors declare no conflicts of interest.

### Peer Review

The peer review history for this article is available at https://publons.com/publon/10.1002/brb3.70389.

## Data Availability

The data that support the findings of this study are available from the corresponding author upon reasonable request.

## References

[brb370389-bib-0001] Chao, B. H. , F. Yan , Y. Hua , et al. 2021. “Stroke Prevention and Control System in China: CSPPC‐Stroke Program.” International Journal of Stroke 16, no. 3: 265–272. 32223541 10.1177/1747493020913557

[brb370389-bib-0002] Dobkin, B. H. 2005. “Rehabilitation After Stroke.” New England Journal of Medicine 352, no. 16: 1677–1684. 15843670 10.1056/NEJMcp043511 PMC4106469

[brb370389-bib-0003] Kam Yuet Wong, F. , S. L. Wang , S. S. Ng , et al. 2022. “Effects of a Transitional Home‐based Care Program for Stroke Survivors in Harbin, China: a Randomized Controlled Trial.” Age and Ageing 51, no. 2: afac027. 35180283 10.1093/ageing/afac027

[brb370389-bib-0004] Tu, W. J. , Z. Zhao , P. Yin , et al. 2023. “Estimated Burden of Stroke in China in 2020.” JAMA Network Open 6, no. 3: e231455–e231455. 36862407 10.1001/jamanetworkopen.2023.1455 PMC9982699

[brb370389-bib-0005] Cieza, A. , K. Causey , K. Kamenov , S. W. Hanson , S. Chatterji , and T. Vos . 2020. “Global Estimates of the Need for Rehabilitation Based on the Global Burden of Disease Study 2019: A Systematic Analysis for the Global Burden of Disease Study 2019.” Lancet 396, no. 10267: 2006–2017. 33275908 10.1016/S0140-6736(20)32340-0 PMC7811204

[brb370389-bib-0006] Feigin, V. L. , B. A. Stark , C. O. Johnson , et al. 2021. “Global, Regional, and National Burden of Stroke and Its Risk Factors, 1990–2019: A Systematic Analysis for the Global Burden of Disease Study 2019.” Lancet Neurology 20, no. 10: 795–820. 34487721 10.1016/S1474-4422(21)00252-0 PMC8443449

[brb370389-bib-0007] Tu, W. J. , Y. Hua , F. Yan , et al. 2022. “Prevalence of Stroke in China, 2013–2019: A Population‐Based Study.” Lancet Regional Health‐Western Pacific 28: 100550. 36507089 10.1016/j.lanwpc.2022.100550 PMC9727498

[brb370389-bib-0008] Tu, W. J. , X. Zeng , and Q. Liu . 2022. “Aging Tsunami Coming: The Main Finding From China's Seventh National Population Census.” Aging Clinical and Experimental Research 34, no. 5: 1159–1163. 34727357 10.1007/s40520-021-02017-4

[brb370389-bib-0009] Asakawa, T. , L. Zong , L. Wang , Y. Xia , and H. Namba . 2017. “Unmet Challenges for Rehabilitation After Stroke in China.” Lancet 390, no. 10090: 121–122. 10.1016/S0140-6736(17)31584-2 28699584

[brb370389-bib-0010] Bettger, J. P. , Z. Li , Y. Xian , et al. 2017. “Assessment and Provision of Rehabilitation Among Patients Hospitalized With Acute Ischemic Stroke in China: Findings From the China National Stroke Registry II.” International Journal of Stroke 12, no. 3: 254–263. 28381197 10.1177/1747493017701945

[brb370389-bib-0011] Ayala, C. , J. Fang , C. Luncheon , et al. 2018. “Use of Outpatient Rehabilitation Among Adult Stroke Survivors—20 States and the District of Columbia, 2013, and Four States, 2015.” Morbidity and Mortality Weekly Report 67, no. 20: 575. 29795076 10.15585/mmwr.mm6720a2 PMC6433337

[brb370389-bib-0012] Kinoshita, S. , M. Abo , T. Okamoto , and K. Miyamura . 2022. “Transitional and Long‐Term Care System in Japan and Current Challenges for Stroke Patient Rehabilitation.” Frontiers in Neurolology 12: 711470. 10.3389/fneur.2021.711470 PMC8786721 35087461

[brb370389-bib-0013] De Peretti, C. , A. Gabet , C. Lecoffre , P. Oberlin , V. Olié , and F. Woimant . 2018. “Regional Disparities in Acute and Post‐Acute Care of Stroke Patients in France, 2015.” Revue Neurologique 174, no. 7‐8: 555–563. 29703444 10.1016/j.neurol.2017.09.014

[brb370389-bib-0014] Phan, H. T. , C. L. Blizzard , M. J. Reeves , et al. 2017. “Sex Differences in Long‐Term Mortality After Stroke in the INSTRUCT (INternational STRoke oUtComes sTudy): A Meta‐Analysis of Individual Participant Data.” Circulation. Cardiovascular Quality and Outcomes 10: e003436. 28228454 10.1161/CIRCOUTCOMES.116.003436

[brb370389-bib-0015] Glader, E. L. , B. Stegmayr , B. Norrving , et al. 2003. “Sex Differences in Management and Outcome After Stroke: A Swedish National Perspective.” Stroke; A Journal of Cerebral Circulation 34, no. 8: 1970–1975. 10.1161/01.STR.0000083534.81284.C5 12855818

[brb370389-bib-0016] Carcel, C. , X. Wang , E. C. Sandset , et al. 2019. “Sex Differences in Treatment and Outcome After Stroke: Pooled Analysis Including 19,000 Participants.” Neurology 93, no. 24: e2170–e2180. 31719135 10.1212/WNL.0000000000008615

[brb370389-bib-0017] Bonkhoff, A. K. , M. D. Schirmer , M. Bretzner , et al. 2021. “Outcome After Acute Ischemic Stroke Is Linked to Sex‐Specific Lesion Patterns.” Nature Communications 12, no. 1: 1–14. 10.1038/s41467-021-23492-3 PMC8172535 34078897

[brb370389-bib-0018] Volpe, S. G. , M. C. Zuniga , M. R. Caunca , and N. Rosendale . 2023. “Gender and Sex Equity in Stroke Research, Education, and Care.” Stroke; A Journal of Cerebral Circulation 54, no. 2: e44–e47. 10.1161/STROKEAHA.122.039893 36689600

[brb370389-bib-0019] McInnes, C. , C. McAlpine , and M. Walters . 2008. “Effect of Gender on Stroke Management in Glasgow.” Age and Ageing 37, no. 2: 220–222. 18006509 10.1093/ageing/afm153

[brb370389-bib-0020] Tu, W. , and W. Wei . 2024. “Trends and Projections in Rehabilitation Demand Across 3 Decades in China (1990− 2019).” Journal of Aging and Rehabilitation 1, no. 2: 31–35.

[brb370389-bib-0021] Asakawa, T. , L. Zong , L. Wang , Y. Xia , and H. Namba . 2017. “Unmet Challenges for Rehabilitation After Stroke in China.” Lancet 390, no. 10090: 121–122. 10.1016/S0140-6736(17)31584-2 28699584

[brb370389-bib-0022] Liu, L. , W. Chen , H. Zhou , et al. 2020. “Chinese Stroke Association Guidelines for Clinical Management of Cerebrovascular Disorders: Executive Summary and 2019 Update of Clinical Management of Ischaemic Cerebrovascular Diseases.” Stroke and Vascular Neurology 5, no. 2: 159–176. 32561535 10.1136/svn-2020-000378 PMC7337371

[brb370389-bib-0023] Ye, Q. , F. Zhai , B. Chao , et al. 2022. “Rates of Intravenous Thrombolysis and Endovascular Therapy for Acute Ischaemic Stroke in China Between 2019 and 2020.” Lancet Regional Health–Western Pacific 21, no. 00: 100406. 35243459 10.1016/j.lanwpc.2022.100406 PMC8873940

[brb370389-bib-0024] Wu, S. , B. O. Wu , M. Liu , et al. 2019. “Stroke in China: Advances and Challenges in Epidemiology, Prevention, and Management.” Lancet Neurology 18, no. 4: 394–405. 30878104 10.1016/S1474-4422(18)30500-3

[brb370389-bib-0025] Chen, H. , Y. Qian , Y. Dong , et al. 2020. “Patterns and Changes in Life Expectancy in China, 1990–2016.” PLoS ONE 15, no. 4: e0231007. 32236129 10.1371/journal.pone.0231007 PMC7112202

